# Pathogenicity and virulence of *Francisella tularensis*

**DOI:** 10.1080/21505594.2023.2274638

**Published:** 2023-11-08

**Authors:** Manon Degabriel, Stanimira Valeva, Sandrine Boisset, Thomas Henry

**Affiliations:** a CIRI, Centre International de Recherche en Infectiologie, Inserm U1111, Université Claude Bernard Lyon 1, CNRS, UMR5308, ENS de Lyon, Univ Lyon, LYON, France; b Univ. Grenoble Alpes, CHU Grenoble Alpes, CNRS, CEA, UMR5075, Institut de Biologie Structurale, Grenoble, France

**Keywords:** Francisella, tularaemia, T6SS, LPS, innate immunity, virulence

## Abstract

Tularaemia is a zoonotic disease caused by the Gram-negative bacterium, *Francisella tularensis*. Depending on its entry route into the organism, *F. tularensis* causes different diseases, ranging from life-threatening pneumonia to less severe ulceroglandular tularaemia. Various strains with different geographical distributions exhibit different levels of virulence. *F. tularensis* is an intracellular bacterium that replicates primarily in the cytosol of the phagocytes. The main virulence attribute of *F. tularensis* is the type 6 secretion system (T6SS) and its effectors that promote escape from the phagosome. In addition, *F. tularensis* has evolved a peculiar envelope that allows it to escape detection by the immune system. In this review, we cover tularaemia, different *Francisella* strains, and their pathogenicity. We particularly emphasize the intracellular life cycle, associated virulence factors, and metabolic adaptations. Finally, we present how *F. tularensis* largely escapes immune detection to be one of the most infectious and lethal bacterial pathogens.

## Brief history of tularaemia

The history of tularaemia and its causative agent, *F. tularensis*, dates back to the early 1900s, when Dr. George McCoy investigated a bubonic plague outbreak in California. The outbreak coincided with a massive die-off of ground squirrels in surrounding areas [1]. Between 1908–1910 McCoy’s team autopsied over 105,000 ground squirrels and identified *Bacillus pestis* (later renamed *Yersinia pestis*) in the samples, confirming the bubonic plague outbreak. However, microbiological smears from some affected squirrels did not reveal the characteristic bacilli of *B. pestis* [2]. When the bacterium was finally purified and stained, it was named *Bacterium tularense* [3], after the county of Tulare in California, one of the areas affected by the epidemic. *B. tularense* was a very small (0.2–0.7 µm) non-motile oval organism surrounded by a clear area likely corresponding to the capsule. The organisms were present in very high numbers in spleen samples from diseased animals and were often found in leukocytes.

The first human case of tularaemia was confirmed in 1914 [4] in a patient with eye inflammation presenting with ulcers and swelling of the eyelid, an infection that progressed to swollen lymph nodes, abscesses, fever, and overall weakness. Later, Dr. Edward Francis investigated cases of a mysterious febrile disease presumed to be transmitted through deer fly bites [5]. He grew a pathogen that he identified as *B. tularense*. Between 1917 and 1919, two dozen cases were reported, one of which was fatal [6]. In the years following, Dr. Francis extensively studied the disease he called tularaemia [6] and its agent, *B. tularense*, gathering data from 14,000 cases by 1944 [7] and himself contracting the disease. He meticulously described each case, cataloguing vectors, routes of infection, symptoms, outcomes, histology, and microbiology of the patient samples [8–10].

Bacteria responsible for tularaemia have been referred to by various names, including *Bacterium tularense, Bacillus tularensis*, *Brucella tularensis*, and *Pasteurella tularensis*. In 1947, Soviet microbiologist Dorofeev proposed the creation of the genus *Francisella* and the species *Francisella tularensis* [11], which is now the accepted name for the causative agent of tularaemia.

Although tularaemia is primarily a natural infection, *F. tularensis* has also been identified as one of the top biowarfare agents. In the 1930s, the Japanese army conducted human tests with *F. tularensis* [12,13]. Around the same time, the US studied *F. tularensis* at a bio-defence research laboratory in Fort Detrick, Maryland, to assess its dangers and develop countermeasures against possible bioterrorism weapons. In 1961, vaccinated and non-vaccinated volunteers were inoculated with *F. tularensis* subcutaneously or through aerosols to test the efficacy of a tularaemia vaccine. The tularaemia respiratory challenge determined that an exposure of less than a minute to 14–15 organisms in 10 litres of air was sufficient to provoke illness in a healthy person [14]. In the 1960s, the US evaluated biological weapon delivery systems and studied the biological decay and dissemination capabilities of *F. tularensis* and other pathogens in natural environments [15]. Former Soviet scientist Ken Alibek claimed that the USSR, and later Russia, were working on bioweapons, including genetically engineered *F. tularensis* strains [16]. Currently, *F. tularensis* is still considered a possible bioterrorism threat in many countries.

## Tularaemia in humans; diseases and associated symptoms

Tularemia is a zoonosis with no inter-human transmission. The infection is usually contracted through direct contact with an infected animal (handling, butchering, or being bitten). Hares or small rodents (voles, mice, rats, coypu, squirrels, etc.) are the primary sources of direct contamination, but over 250 animal species have been identified as carrying or being infected with *F. tularensis* [17]. Most infected animals are accidental hosts, not true reservoirs, but contribute to the pathogen’s life cycle [18].

Tularaemia in humans presents in six clinical forms depending on the point of bacterial entry: ulceroglandular, glandular, oculoglandular, oropharyngeal, pulmonary, and typhoid. At onset, tularaemia symptoms are non-specific and include a flu-like syndrome with fever, headache, muscle and joint aches (myalgia, and/or arthralgia). Later, one or more chronically evolving lymphadenopathies can develop in the lymphatic system draining the site of entry.

Ulceroglandular and glandular forms are the most common, accounting for 60–70% of tularaemia cases. Ulceroglandular forms are characterized by an inoculation ulcer with one or more satellite lymphadenopathies. The glandular forms of the tularaemia do not have an identified ulcer. Despite appropriate treatment, approximately 30% of patients with adenopathy progress to lymph node abscesses requiring surgical removal [19]. Ulceroglandular and glandular forms also occur after bites from haematophagous arthropods (e.g. ticks, mosquitoes, and horse flies) or contact with a contaminated environment during outdoor leisure activities (e.g. gardening, swimming in rivers or lakes, and canyoning) [20,21]. Tick bites are the primary source of tularaemia transmission in the US, and a significant source in Europe [22,23].

The oculoglandular forms are rare and occur through manual transmission of bacteria to the ocular sphere or by contaminated droplets. They cause painful conjunctivitis and swelling around the eye (palpebral oedema) and are associated with enlargement of the lymph nodes localized near the ear or neck (periauricular or cervical lymph nodes, respectively). Oropharyngeal forms result from food contamination by carcasses or animal excrement and present with pharyngitis, digestive disorders, and one or more cervical lymphadenopathy [20].

Pulmonary tularaemia results from airborne infections through infectious aerosols or contaminated dust from various activities (lawn mowing, sweeping a barn, handling hay, etc.) [24]. Symptoms include cough, chest pain, rapid respiration (polypnea), fever, and enlargement of lymph nodes localized in the chest region (mediastinal and/or hilar adenopathy) [20]. The typhoidal or septicaemic form is a systemic form that accounts for approximately 10% of the cases. It has no detected portal of entry or adenopathy, but patients exhibit fever, fatigue, and sometimes neurological and/or digestive disorders (vomiting, diarrhoea, and abdominal pain). Typhoidal tularaemia is usually diagnosed by isolation of the bacteria in blood culture and could represent a bacteraemia phase of tularaemia in some patients before the appearance of adenopathy [20]. The pulmonary and typhoid forms are the most severe forms of tularaemia and are potentially fatal. The fatality rate of tularaemia is less than 1% to 3–5%, in Europe and the US, respectively, but it can be as high as 30% in the pulmonary form [20]. The prognosis of tularaemia depends on several factors, including the clinical form, patient’s condition, time to initiate effective antibiotic therapy, and subspecies or clade infecting the patient (see below).

### Francisella genus

The *F. tularensis* species (Table 1) includes three subspecies, each distinguished by its metabolic characteristics and virulence: *F. tularensis* subsp. *tularensis*, *F. tularensis* subsp. *holarctica* and *F. tularensis* subsp. *mediasiatica*. Two of these subspecies, *tularensis* (type A strains) and *holarctica* (type B strains), are responsible for human cases of tularaemia [25], with the former found only in North America and the latter throughout the Northern Hemisphere. The third subspecies, *mediasiatica*, is present in Central Asia but has never been isolated from humans. In addition, the environmental species *F. novicida* sometimes appears as a fourth subspecies (Table 1), and the two names *F. novicida* and *F. tularensis* subsp. *novicida* coexist in the literature [31]. *F. novicida* has lower metabolic requirements than *F. tularensis* and shares over 97% genome homology with *F. tularensis* [32] (Table 2). *F. novicida* appears to have only an aquatic reservoir because it has never been isolated from arthropods or small animals. They have a low virulence in humans. Indeed, rare human infections with *F. novicida* have been reported, mainly in immunocompromised patients, after exposure to contaminated water [33].

**Table 1.** Reservoir, vectors and pathogenicity for humans of different species of *Francisella*..

Species or subspecies of Francisella	Reservoir/vectors	Pathogenic for humans	References
*F. tularensis subsp tularensis*	More than 250 animal species, including lagomorphs and small rodents Ixodidae ticks, mosquitoes and deer flies Contaminated environment (soil, water.)	yes	[25]
*F. tularensis subsp holarctica*	More than 250 animal species, including lagomorphs and small rodents Ixodidae ticks, mosquitoes and deer flies Contaminated environment (soil, water.)	yes	
*F. tularensis subsp mediasiatica*	Ticks	No	[28]
*F. novicida*	Sea- water brackish-water	Occasionally (immunocompromised patients, neardrowning accident)	
*F. hispaniensis*	Poorly characterized Aquatic sources … .	Rarely	
*F. persica*	Ticks	No	[26]
*F. opportunistica*	environment	Rarely immunocompromised patients	[27]
*F. philomiragia*	salt- or brackish-water sick muskrat Dermacentor ticks	Occasionally (near-drowning accident, immunocompromised patients -especially people suffering from chronic granulomatous disease)	
*F. noatunensis* *F. noatunensis subsp. orientalis* *F. noatunensis subsp. noatunensis*	warm- and cold-water fish pathogens	No	[29]
*F. salimarina/F. marina/F. salina*	Sea-water Fish pathogens	Very rarely (one case described)	[29]
*F. endocilophora*	endosymbionts of marine ciliates	No	[29]
*F. halioticida*	Pathogen for molluscs	Very rarely (one case described)	[30]
*F. uliginis*	sea-water	No	[29]

**Table 2.** Characteristics of *Francisella* subspecies traditionally used to study virulence.

	*F. novicida*	*F. tularensis subsp tularensis*	*F. tularensis subsp holarctica*	References
Reference strains	U112	SCHU S4	LVS	
Genome size	1,910,031 bp	1,892,819 bp	1,895,998 bp	[33]
Protein coding genes	1731	1145	1380	[33]
Sequence identity to SCHU S4	98.1%	/	99.2%	[34]
FPI	1 copy	2 copies	2 copies	[35]
Type VI secretion system effectors	OpiA, OpiB1–3, pdpD, pdpC	Splitted OpiA in 2 ORF, small OpiB*, pdpD, pdpC	Splitted OpiA in 2 ORF, small OpiB**, pdpC	[35–37]

*Ankyrin repeat region is absent.

**Ankyrin repeats region absent and deletion in the C-ter region.

In this review, the term *Francisella* refers to both *F. tularensis* and *F. novicida*, while *F. tularensis* and *F. holarctica* refer to their respective subspecies. Most of the knowledge in the field comes from the study of *F. novicida* strain U112, *F. tularensis* strain SCHU S4, and *F. holarctica* strain LVS (Table 2). The latter, which stands for Live Vaccine Strain, is a live attenuated strain obtained through serial passages that demonstrated some immunization efficacy against SCHU S4 challenge in humans. However, to date, neither LVS nor any other strains are currently licenced as vaccine strains for tularaemia [38].

Some other *Francisella* species can be pathogenic to humans in rare cases (Table 1). *F. philomiragia* is a predominantly waterborne reservoir species with low virulence, but rare cases of human infections linked to this bacterium have been described in immunocompromised patients, notably during chronic septic granulomatosis [39]. *F. hispaniensis* [40] and *F. opportunistica* [41] have also been described as responsible for human infections.

Several other species of *Francisella* exist, but they have not yet been identified as agents of human infection. Some are pathogens of fish or molluscs, such as *F. orientalis* [42], *F. noatunensis* [43], *F. halioticida* [44] and *F. marina* [45], whereas others are isolated from aquatic environments, such as *F. salina* [46], *F. uliginis* [46] and *F. salimarina* [47].

### Infection process-lessons from animal models

*Francisella* causes different diseases depending on the route of entry. To study these diseases, several mouse models of tularaemia have been developed, including ulceroglandular and pneumonic tularaemia.

Following inhalation, *Francisella* is found mostly in alveolar macrophages 24 h post-infection. By day three post-infection, 50–80% of the infected cells are neutrophils (for SCHU S4 and *F. novicida* U112 strains, respectively) [48]. Regardless of the route of infection, *Francisella* quickly disseminates to various organs, including the spleen and the liver. While phagocytes are thought to be the primary cell type hosting *Francisella*, *Francisella* can also replicate in other cell types such as alveolar epithelial cells [49] and hepatocytes [50]. These cells could become important reservoirs at late time points of infection.

In the liver, infection with the LVS strain leads to rapid formation of granulomas that spatially restrict infection [51]. In the murine model, SCHU S4 infection leads to rapid death (within 5 days), making it difficult to study adaptive immune responses unless an appropriate antibiotic regimen is provided [52]. Depending on the route of entry, *F. novicida* infection can be highly (LD50 ~ 10) or moderately lethal to mice following intranasal or intradermal inoculation, respectively [33]. The difference in LD50 between the intradermal and the intranasal routes is poorly understood. The kinetic of bacterial dissemination to systemic organs is clearly longer upon intradermal than upon intranasal inoculation. The Intradermal injection may promote the establishment of an efficient antibacterial IFN-γ-producing immune response. In contrast, the immune response upon intranasal inoculation appears outcompeted at systemic sites by the rapid bacterial replication, leading to massive inflammation contributing to tissue damage [53].

## Intracellular life cycle (Fig. 1)

### Entry

As described above, *F. tularensis* primarily replicates in phagocytes. Hence, bacteria enter cells by phagocytosis to reside in a *Francisella*-containing phagosome (FCP). The engagement of different phagocytic receptors, such as the mannose receptor, scavenger receptor A, complement receptor CR3, or FcγRs [54–59] depends on the host’s immune status, especially the presence of opsonizing antibodies. Opsonization through either antibody or complement deposition strongly enhances *F. tularensis* phagocytosis. The route of entry also affects the trafficking of the FCP, the efficiency of phagosome escape, the pro-inflammatory response, and ultimately the infection outcome [54,60]. In particular, IgG-opsonized *F. tularensis* escapes from the phagosome less frequently than does a non-opsonized bacterium. IgG-opsonized *F. tularensis* is also highly restricted in its cytosolic replication in a phagosomal NADPH oxidase-dependent manner [54]. Natural IgM recognizing *F. tularensis* capsule and/or O-antigen can also facilitate *F. tularensis* entry by initiating the complement cascade and promoting CR3-dependent phagocytosis [61].

**Figure 1.**
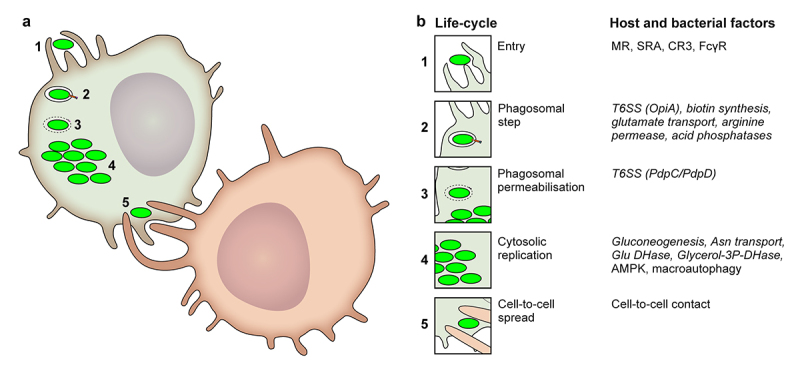
*Francisella* life cycle and the main host and bacterial factors involved at each step.

(a) Schematic representation of *Francisella* life cycle in macrophages. Merocytophagy of uninfected macrophages (red) is shown in panel 5. (b) For each step in the life cycle, the main host and *bacterial* factors (in italics) are shown.

### Early trafficking events and phagosomal escape

The kinetics of marker accumulation on the phagosome are consistent with the progressive maturation of the FCP along the endocytic pathway. Early on, within less than 5 min post-entry, FCP accumulates the early endosome marker EEA1. This marker is then lost when the late endosomal/lysosomal marker LAMP-1 is gained [62]. Depending on the studies [62–64], the frequency of LAMP-1^+^ FCP/intact phagosomes peaks between 20 min and 4 h post-infection, corresponding to the kinetics of *Francisella* escape into the cytosol. This escape is dependent on several genes clustered on a genomic island known as the *Francisella* Pathogenicity Island (FPI) [63,65–69]. The FPI, FPI-encoded type VI secretion system (T6SS), its effectors, and their role in FCP maturation and *Francisella* escape into the host cytosol are described in detail below.

Despite *Francisella*’s rapid escape from the phagolysosome, several features are important for its short transit through the acidified phagosome and the ensuing bacterial escape. Biotin synthesis is required for phagosomal escape [70] as it is necessary for growth in nutrient-limiting environments. This suggests that nutritional adaption to the phagosome is key to escape. In agreement with this hypothesis, an *F. novicida ΔgadC* mutant lacking a glutamate transporter is deficient in vacuolar escape [71]. Growth of the *ΔgadC* mutant is restored in macrophages deficient for NADPH oxidase, suggesting that glutamate and the tricarboxylic acid (TCA) cycle in which glutamate is utilized are important for resisting the transient oxidative burst that occurs in the FCP [71]. Notably, glutamate assimilation is also important for carbon acquisition in the host cell cytosol [72] (see below). Additionally, the arginine permease ArgP [73] (and, to a lesser extent, the isoleucine permease [74] is required for *Francisella* to escape into the cytosol. In addition, four acid phosphatases (AcpA, AcpB, AcpC, and HapA) have been reported to be required for *F. novicida* phagosomal escape [75] and to contribute to *F. tularensis* SCHU S4 escape [76] likely by opposing phagosomal NADPH oxidase assembly and reactive oxygen species production [77]. Interestingly, these studies point towards an important role for NADPH oxidase in blocking phagosomal escape. *F. novicida* resistance to oxidative stress is also mediated by the lysine decarboxylase LdcF, possibly through the generation of cadaverine which scavenges oxygen radicals [78]. Finally, the generation of ammonia by deaminases buffers the phagosomal pH [79]. Notably, citrulline ureidase, present in type A strains (e.g. SCHU S4) but absent in type B (*F. tularensis spp. holarctica*), degrades citrulline to ornithine and ammonia and contributes to bacterial growth in IFN-γ-treated macrophages [80]. Alternatively, by diverting citrulline catabolism from L-arginine to ornithine, the citrulline ureidase may reduce L-arginine availability. L-arginine is the substrate of IFN-γ-inducible NO synthase (iNOS), hence the citrulline ureidase may allow *F. tularensis* type A strain to limit NO production in infected macrophages. Accordingly, the growth of the citrulline ureidase mutant was not reduced compared to that of WT SCHU S4 in iNOS^KO^ macrophages [80].

### Cytosolic replication

After escaping phagosomes, *Francisella* resides in the nutrient-rich cytosol of the host cell, which is equipped with innate immune sensors (see below).

The bacterium adapts its metabolism to the cytosolic environment by relying on gluconeogenesis and amino acid catabolism as carbon and energy sources. In contrast, glycolysis is dispensable [72,81]. Indeed, GlpX, a fructose 1, 6-bisphosphatase enzyme required for gluconeogenesis, is crucial for *Francisella* growth in the macrophage cytosol, as mutants lacking *GlpX* do not replicate unless the medium is supplemented with glucose [72,81]. *Francisella* also utilizes asparagine and glutamate as carbon sources through the asparagine transporter (AnsP) and glutamate dehydrogenase (GdhA), respectively [82]. The catabolism of these two amino acids feeds into the TCA cycle to provide the energy required for replication. In addition to relying on amino acids as a source of carbon and nitrogen, *Francisella* genomes encode a glycerol uptake and a glycerol-3 phosphate transporter, and thus likely use pyruvate, glycerol, and glycerol-3P as carbon sources to sustain its replication within the host cytosol [83]. Accordingly, glycerol-3P dehydrogenase (GlpA) is required for *Francisella* replication [72]. Lipolysis (oxidation of fatty acids to generate glycerol) occurs within infected cells and is a process regulated by the activation of the host regulatory kinase AMPK. Inhibition of lipolysis or knockout of *AMPK* strongly reduces *Francisella* replication, likely due to the decreased production of glycerol and glycerol-3P [72,84]. Importantly, *Francisella* spp. are auxotrophs for at least 9 different amino acids [85], and 13 amino acids were found to be essential to sustain *Francisella* growth in a chemically defined growth medium [86]. These observations indicate that importing amino acids from the host cytosol is key to sustaining virulence. The assimilation of one amino acid, cysteine, has been particularly studied and shown to rely on the import of host glutathione (GSH, a tripeptide γ-L-glutamyl-L-cysteinyl-glycine) [87–89].

These metabolic requirements clearly indicate that *Francisella* needs to scavenge a large quantity of nutrients to sustain its intracellular replication. Macro-autophagy, a pathway leading to the degradation of organelles and cytoplasmic content, promotes *Francisella* intracytosolic replication [90]. Inhibition of autophagy impairs *Francisella* replication unless the cell culture medium is supplemented with amino acids or pyruvate. Thus, *Francisella* utilizes macroautophagy as a process to generate sufficient essential carbon and energy sources from the macrophage cytosol. Interestingly, this process is independent of the canonical autophagy protein Atg5 [90]. Atg5-dependent xenophagy (a specific macro-autophagy process targeting intracellular pathogens) is usually an antibacterial mechanism that leads to the capture and degradation of cytosolic bacteria [91]. However, wild-type *Francisella* strains can escape atg5-dependent xenophagy [92,93]. In contrast, mutants of genes implicated in LPS O-antigen synthesis or strains unable to replicate within the host cytosol (e.g. purine auxotrophs) are targeted by atg5-dependent xenophagy process [92]. Remarkably, myeloid-specific *atg5*^KO^ mice are more resistant to LVS infection than WT mice, emphasizing that WT *Francisella* benefits from autophagy responses [94].

Another layer of complexity in the interactions between *Francisella* and autophagy lies in the formation of *Francisella*-containing vacuoles (FCVs). FCVs are observed at late time points of infection in primary murine bone marrow-derived macrophages but are absent in infected human monocyte-derived macrophages [62]. These large vacuoles, whose formation is blocked by 3-methyladenine (3-MA, an autophagy inhibitor), require cytosolic replication of *Francisella*, contain a large number of bacteria, are positive for both autophagy markers (e.g. LC3), and interact with late endocytic/lysosomal compartments. The outcome of *Francisella* enclosed within these FCP remains unknown, although bacteria appear intact [62].

### Cell to cell spread

In contrast to most cytosolic pathogens [95], *Francisella* is devoid of actin-based motility, which is one of the mechanisms used by other pathogens to disseminate from cell to cell. *Francisella* may escape from infected cells after cell death, which often occurs when the cells are filled with bacteria. However, at least in the case of pyroptosis, cell death might trap bacteria inside host cell remnants, leading to their elimination by efferocytosis (removal of dead cells) [96]. Another mechanism of cell-to-cell transfer, termed merocytophagy (“mero,” Greek for partial; “cytophagy” for cell eating), has been observed during cell-to-cell contact [97]. During this process, uninfected macrophages come in contact with infected cells and phagocytose portions (membrane and cytosolic materials) of neighbouring cells. Whenever these phagocytosed bits include cytosolic *Francisella*, the “eating” macrophages become infected. Importantly, in this merocytophagy process, the engulfed *Francisella* is found in a double-membrane vacuole and require T6SS and its effectors to escape into the cytosol of the newly infected cell [97]. Compared with the uptake of extracellular bacteria, this mode of cell-to-cell transfer appears to enhance transmission, at least *in vitro*.

### Francisella virulence factors

#### The central virulence device: F. tularensis T6SS

In 2002, transposon mutagenesis was used in *F. novicida* to identify two ORFs in a four-gene operon as being required for intracellular replication [69]. The four genes were named *IglA-D* for “intracellular growth locus.” This locus was later found to belong to a larger locus of ~ 30 kb containing 16–19 ORFs, with G+C content deviating from the rest of the genome, and hence named FPI for “*Francisella* Pathogenicity Island” [98]. The genes within the FPI were named *iglA-J* (cf above) and *pdpA-E* as “pathogenicity-determinant proteins.” IglC, previously found to be one of the main bacterial proteins induced during host cell infection [99], was identified to be required for *Francisella* escape from the phagosome in 2004 [66]. Early studies [67,69,98,100] suggested that FPI may encode a secretion system, and in 2009, FPI was demonstrated to encode a Type VI Secretion System (T6SS) [63]. *F. tularensis* and *F. holarctica* contain two almost identical copies of FPI. These two copies may be functionally redundant, as deletion of only one FPI copy does not affect the virulence of *F. tularensis*, in stark contrast to the removal of both FPI loci [101]. Interestingly, *F. novicida* contains only a single copy of FPI, but another genomic island termed “*Francisella novicida* Island” (FNI) [32,102,103] is present in the *F. novicida* genome. FNI presents some similarity with FPI, suggesting that it might encode another T6SS [104]. FNI is not involved in *F. novicida* virulence in a mouse model of tularaemia [102].

T6SSs are widespread among Gram-negative bacteria. They belong to the family of contractile injection systems (CISs), a needle-like structure that shares 13 or 14 core components, and is implicated in the secretion of effectors from the bacterial cytosol into target cells. Evolutionarily divergences in terms of structure, function, or gene content allow the classification of four subtypes [105]. *Francisella* T6SSs are the only representatives of the second subtype of T6SS, T6SS^ii^, and show structural and functional homologies to canonical T6SS (T6SS^i^) components [106].

*Francisella* T6SS is required for phagosomal escape, intracytoplasmic replication in host cells, and virulence in animals [107]. Deletion of FPI fully abolishes *Francisella* escape into the host cytosol and its virulence in mice and in *Galleria mellonella* larvae [65,103,108].

Six proteins have been characterized in *F. novicida* as T6SS effectors based on an *in vitro* secretion assay: PdpC, PdpD, OpiA, OpiB1, OpiB2, OpiB3 [36]. PdpD is absent in *F. holarctica* [98]. Furthermore, in both *F. holarctica* and *F. tularensis*, OpiA is split into two ORFs, and a single OpiB paralog, devoid of the ankyrin repeats found in *F. novicida* OpiB1–3 [36,37], is present. PdpD and PdpC are the two key effectors required for phagosomal escape of bacteria into the host cytosol. While deletion of either *pdpC* or *pdpD* has only a minor impact on phagosomal escape, an *F. novicida* double *ΔpdpCΔpdpD* mutant is fully deficient in its ability to reach the cytosol [109]. Surprisingly, in LVS (which is naturally deficient in PdpD), a Δ*pdpC* mutant, which is unable to reach the host cytosol, lies in damaged phagosomes, suggesting that other effector(s) might impact phagosomal membrane integrity [110]. The molecular modes of action of PdpC and PdpD remain unknown.

OpiA, OpiB1, OpiB2, and OpiB3 are encoded outside the FPI (hence their names Opi standing for “Outside Pathogenicity Island”). OpiA is a phosphatidylinositol 3-kinase that phosphorylates phosphatidylinositol to generate the glycerophospholipid phosphatidylinositol(3)-phosphate (PI(3)P) on the external leaflet of the FCP. The presence of PI(3)P on the FCP delays its maturation along the endosomal pathway and enhances the intracellular survival of *Francisella* by providing sufficient time for other *Francisella* effectors (e.g. PdpC and PdpD) to act and promote escape into the host cytosol [37]. The contribution of OpiA to the overall virulence of *Francisella* appears to be minor, and has only been demonstrated in a *ΔpdpC* background. No virulence defects have been observed in a *ΔopiB1–3* mutant, and the function of OpiB effectors remains to be discovered [36].

Several studies using various techniques, such as microinjections [111], functional transcomplementation [112] and inducible complementation [97] indicate that T6SS is not required for replication in the host cytosol, suggesting that its primary function is to promote phagosomal escape.

**Figure 2.**
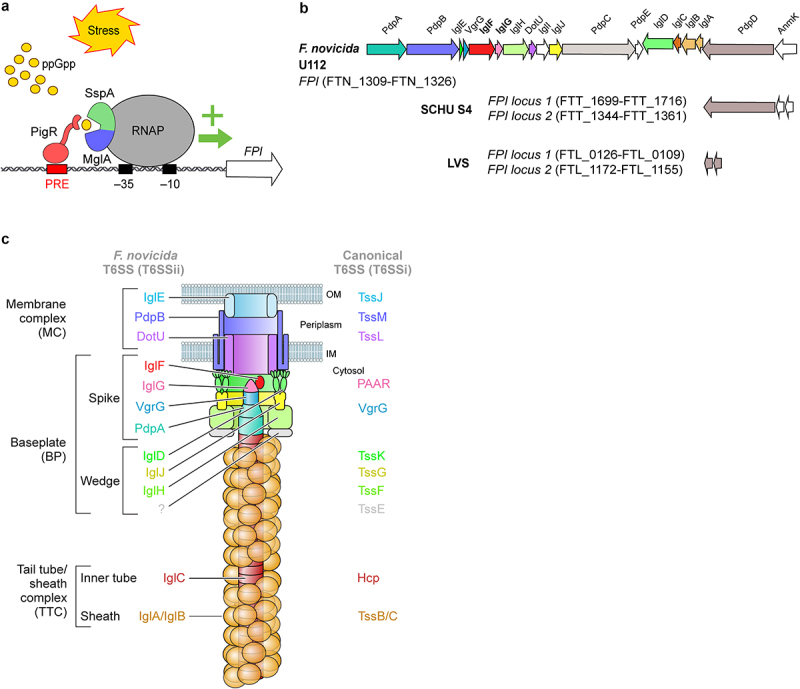
*Francisella* pathogenicity island, its transcriptomic regulation and its encoded T6SS.

The structure-function of the *Francisella* T6SS machinery involved in effector secretion is starting to be well understood (Figure 2) following a series of very elegant studies [36,104,109,113–115] and following the overall advances in the T6SS field [116]. Whenever relevant, we will extrapolate the results from T6SS^i^ studies to propose hypotheses regarding *Francisella* T6SS structure and function.

(a) Transcriptomic regulation of FPI gene transcription (see text for details). (b) Structure of *F. novicida* FPI. The main differences with SCHU S4 and LVS FPI are shown. The genes encoding structural components are in colour (see C), the genes encoding effectors are in grey and the genes encoding proteins are unknown functions in white. (c) Structural model of *Francisella* T6SS with *Francisella* protein name on the left and the name of their T6SS^i^ homologues on the right.

The T6SS machine can be subdivided into i) a membrane complex that anchors the T6SS to the cell envelope, ii) a baseplate composed of a wedge attached to a spike, which is the central organizer of the T6SS, and iii) a tail tube/sheath complex that includes a contractile sheath and an inner tube. The inner tube is capped by the spike complex, and sheath contraction promotes expulsion of this tube/spike complex through the transenvelope membrane complex and into the target cell.

The membrane complex of *Francisella* T6SS is thought to be composed of three components: IglE, PdpB, and DotU. IglE is an outer membrane lipoprotein that shares structural homology with TssJ [117–119] (The Tss nomenclature refers to T6SS^i^ machinery core proteins [120]). The periplasmic domain of IglE interacts with inner membrane PdpB, which shares homology with TssM. In addition, PdpB interacts with the inner membrane protein, DotU/TssL [118,121,122]. IglE, PdpB and DotU are required for *Francisella* T6SS function [109] and although the structure of the IglE/PdpB/DotU complex remains to be determined, we can hypothesize that it is closely similar to the TssJML membrane complex. In *Escherichia coli*, the assembly of the membrane complex occurs first and is independent of other components. TssJLM complex forms a double-ring, allowing the passage of the T6SS inner tube [123].

In *E. coli* T6SS, parallel to the assembly of the membrane complex, a baseplate is assembled in the cytosol and acts as a scaffold onto which the whole T6SS is assembled. The baseplate is composed of TssE, TssF, TssG, and TssK proteins forming the wedge complex, and of the proteins composing the spike (VgrG and PAAR proteins in *E.coli*). *Francisella* wedge complex is not well described, but IglH, IglJ, and IglD are likely homologues of TssF, TssG, and TssK, respectively [113]. Notably, IglD has a similar 3D structure to that of TssK [113]. No TssE homologs have been identified in *Francisella*. In T6SS^i^, the spike complex includes a trimer of valine-glycine protein G (VgrG) proteins that are closely related to the T4 contractile bacteriophage tail spike proteins gp27-gp5 and, when present, a PAAR protein that caps and sharpens the spike complex [124]. *Francisella* VgrG is much smaller than its T6SS^i^ homologues and encodes only the gp5-like domain. VgrG interacts with PdpA and PdpA structure is partially homologous to gp27. The (VgrG-PdpA)_3_ complex forms the central spike of *Francisella* T6SS and demonstrates unique features, including the presence of a basal lid of unknown function and a cavity that might accommodate a small effector [36,115]. IglG was identified by homology modelling as a PAAR-like protein [104] and is now classified as belonging to the PAAR-H subtype [125]. Although IglG is thought to cap *Francisella* T6SS, an interaction between IglG and VgrG has not been demonstrated. IglG interacts with another FPI protein of unknown function, IglF. In T6SS^i^, one of the main mechanisms of effector secretion is associated with spike proteins. The mechanism underlying effector secretion in *Francisella* remains unexplored.

The baseplate interacts with the membrane complex, and the other side acts as a scaffold to polymerize the tail tube/sheath complex. Indeed, PdpA interacts with IglC, an Hcp homologue [68]. Hcp proteins homopolymerize to form hollow hexameric rings that are stacked on each other to form an inner tube. Some effectors are present within the Hcp tube and secreted together with the tube. However, this mechanism has not yet been described in *Francisella*. The Hcp tube promotes the assembly of the contractile sheath, which is composed of a helix of the TssB/C heterodimer. *F. novicida* T6SS sheath was purified and visualized by cryoelectron microscopy and was demonstrated to be composed of polymerized IglA/B heterodimers [114].

The contraction of the sheath results in secretion of the inner tube capped by the spike complex and loaded with effectors [116]. Accordingly, in an *in vitro* secretion assay, IglC, PdpA, VgrG, and the six effectors described above were identified in the supernatant [36]. It remains unclear whether IglG and IglF were not secreted or detected, and whether additional effectors remain to be identified.

Recycling of the contracted TSS6^i^ sheath is mediated by unfoldase ClpV, which is missing in *Francisella* and is functionally replaced by ClpB. Indeed, ClpB is required for the disassembly of the IglA/B contracted sheath and likely promotes the recycling of T6SS^ii^ components to promote multiple firing events [109,126]. Interestingly, the function of ClpB as a heat shock response protein can be dissociated from its function as a T6SS^ii^ unfoldase, indicating that in *Francisella* ClpB is a moonlighting protein [127].

#### Regulation of T6SS gene transcription and T6SS assembly

FPI gene expression is induced during macrophage infection. In *Francisella tularensis* SCHU S4, FPI gene expression reaches its highest expression 12-16 h post-infection, corresponding to the intra-macrophage replication phase of the bacterium [128]. The activation of the FPI cluster is controlled by a complex transcription regulatory system that includes stringent starvation protein A (SspA) and macrophage growth locus protein A (MglA) [129–131]. The heterodimer SspA/MglA is constitutively associated with RNA polymerase (RNAP) and stabilizes the σ [79] subunit of RNAP [132]. However, to induce FPI gene expression, two other players are needed: the pathogenicity island regulator (PigR [133] also termed FevR [134]) and the alarmone (p)ppGpp, which is produced upon stringent stress response (a stress response triggered by several cues including amino acid starvation). (p)ppGpp binds to the SspA-MglA complex and promotes the high affinity binding of PigR to SspA-MglA complex [132,135,136]. PigR recognizes a 7bp motif named PigR response element (PRE) located in promoters, including those of *iglA* and *pdpA* [137]. In the presence of (p)ppGpp, PigR via its tight association with SspA-MglA recruits the SspA-MglA-RNAP complex to PRE-containing promoters to drive transcription initiation and ultimately FPI genes transcription. PigR transcription is itself positively regulated by MglA [134], and by the histone-like HU protein [138], which among numerous targets binds the *pigR* promoter [139].

Factors other than stringent stress induce T6SS expression. Indeed, IglC is induced during iron restriction, a condition that is associated with the intracellular environment [140].

Finally, another layer of regulation controls the T6SS assembly. Indeed, specific cues, such as the presence of high potassium concentration (as revealed by culture in medium containing 5% KCl) or specific oxygen tension (which is modified when *F. novicida* is grown on an agarose pad covered with a coverglass) are needed to induce T6SS assembly [109,114]. The mechanism underlying this regulation of T6SS assembly and whether T6SS sheath contraction is itself under specific regulation remains to be discovered, but may implicate post-translational modifications [141].

#### Francisella envelope and innate immune responses

As an intracellular pathogen, *Francisella* is exposed to several innate immune sensors and immune effector mechanisms. *Francisella* is considered a stealth pathogen [142–144] largely because of the properties of its envelope, including the peculiar LPS.

#### Francisella LPS

*Francisella* LPS structure is largely conserved between *F. tularensis* and *F. novicida* (although small differences exist in the O-chain moiety) [145]. Thus, it is expected that most studies performed on one (sub)species will prove to be true for other (sub)species of *Francisella*.

While the prototypical LPS from enterobacteria contains a Lipid A part with six acyl chains (12–14 carbon atoms per chain), *Francisella* Lipid A is tetra-acylated, possesses long acyl chains (16–18 carbons), and is hypophosphorylated. These characteristics are key to evading the innate immune response. Indeed, *Francisella* Lipid A is recognized neither by Toll-Like Receptor 4 [146] nor by the murine intracytosolic LPS sensor, Caspase-11 and only poorly by its human counterpart Caspase-4 [147].

The lack of TLR4 activation results from multiple layers of evasion. Indeed, *Francisella* LPS evades detection by CD14, the most upstream event in the CD14/MD2/TLR4 signalling cascade. In addition, hexacylation is required to trigger dimerization of the TLR4/MD-2 complex and downstream signalling, suggesting that *Francisella* LPS would similarly not trigger this event [148]. Accordingly, innate immune responses against *Francisella* are mainly driven by TLR2 [149–153] which recognizes lipoproteins [154]. *Francisella* has evolved strategies to downregulate lipoprotein expression and TLR2-mediated responses [155,156]. Indeed, *F. novicida* uses the CRISPR/cas9 system to regulate lipoprotein expression (FTN_1103) and evades TLR2 recognition. Interestingly, Cas9 and the associated tracrRNA and scaRNA are induced by a 100 fold during infection, which correlates with the downregulation of FTN_1103 [156]. Surprisingly, in contrast to *F. novicida*, *F. tularensis* has a deficient CRISPR/Cas9 system and is still able to limit TLR activation. This observation suggests that highly virulent *Francisella* species have evolved different mechanisms to downregulate bacterial lipoprotein expression/recognition.

**Figure 3.**
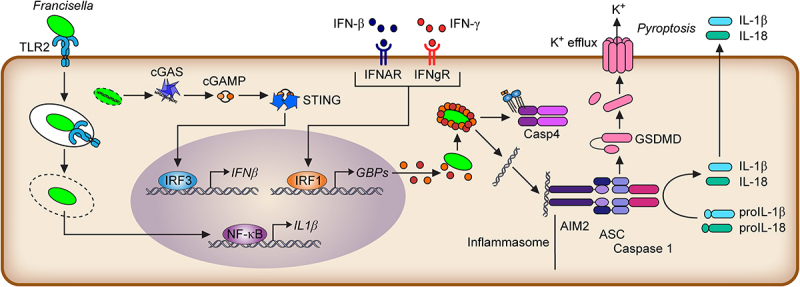
Overview of the innate immune responses upon macrophage infection.

*Francisella* Lipid A largely evades recognition by caspase-11 and non-canonical inflammasomes in murine macrophages. Caspase-11 is a cytosolic Lipid A receptor that dimerizes and is activated by Lipid A binding. Active caspase-11 cleaves Gasdermin D, releasing its N-terminal pore-forming domain that oligomerizes into the plasma membrane and triggers rapid inflammatory cell death, termed pyroptosis (Figure 3). Evasion of caspase-11 recognition is due to its low acylation level. Indeed, Lipid A purified from the *Francisella LpxF* mutant is pentacylated and activates caspase-11 [157]. Caspase-4 is more reactive to *Francisella* LPS (although ∽ 10 times less than to *E. coli* LPS) and under-acylated lipid A [147] than its murine homologue caspase-11. Accordingly, the caspase-4 inflammasome detects *F. novicida* in human macrophages [147].

*Francisella* escapes TLR4 recognition and is recognized by TLR2, which leads to NF-κB activation. Early detection of nucleic acids by cGAS leads to the production of the second messenger cGAMP for STING activation, resulting in IRF3 activation. IFN-β or IFN-γ induces guanylate binding protein (GBPs) production in an IRF1-dependent manner. GBPs cover *Francisella* and promote Caspase-4 recognition of *Francisella* LPS in human macrophages, DNA release into the host cytosol, and activation of the Aim2 inflammasome in murine macrophages culminating in GasderminD (GSDMD) cleavage, pore formation, cytokine secretion, and pyroptosis. T.F.: transcription factors.

The inner core of *Francisella* LPS also displays immunoevasive properties. Indeed, while most Gram-negative bacteria contain heptose residues in the LPS inner core, *Francisella* LPS core is relatively small and consists mainly of two mannose residues [158]. Importantly, ADP-heptose is an intermediate metabolite in the LPS biosynthetic pathway of bacteria containing heptose residues in their core but is also a potent activator of NF-kB. Indeed, the host cytosolic receptor ALPK1 binds ADP-heptose, leading to the activation of its kinase activity and triggering TIFA-TRAF6-dependent activation of NF-kB. Although not experimentally proven, owing to its atypical LPS core, this innate immune pathway should not detect *Francisella* infection [159].

In addition, *Francisella* LPS has other atypical features, including a single 2-keto-3-deoxy-D-manno-octulosonic acid (Kdo) residue in its inner core (whereas LPS from most Gram-negative bacteria displays two or three Kdo residues) [158]. Deletion of the two-component Kdo hydrolase (KdhAB) responsible for this unique feature leads to severe attenuation of the corresponding *Francisella* mutant [160]. Furthermore, over 70% of its lipid A is present as “free lipid A,” which is not attached to the classical core and O-antigen sugar residues [161]. Finally, *Francisella* Lipid A is hypophosphorylated due to the action of the two enzymes LpxE [162]and LpxF [163] that hydrolyse lipid A phosphate residues. The *lpxF* mutant is highly attenuated for virulence [164,165] suggesting that hypophosphorylation is important for virulence. In agreement with the reduction in negative charge associated with the lack of a phosphate group on Lipid A, WT *Francisella* is highly resistant to cationic antimicrobial peptides, a resistance that is lost in the *lpxF* mutant [165]. Notably, since LPS from the *lpxF* mutant displays penta-acylated Lipid A, it remains difficult to conclude specifically on the role of hypophosphorylation in virulence. Similarly, the NaxD enzyme neutralizes the negative charge of a phosphate group present on free lipid A by the addition of galactosamine, which also contributes to cationic antimicrobial peptide resistance [166].

#### Capsule

A capsule-like structure was observed around *F. tularensis* in 1977 [167,168] and later characterized as a capsule of polysaccharide identical to the O-antigen subunit of LPS [169]. The purified capsule lacked other LPS components, suggesting that it is a different entity. Unfortunately, to date, there is no convincing genetic way to invalidate the capsule without affecting LPS O-antigen, rendering the characterization of the capsule and its roles in virulence highly challenging [167].

#### Membrane phospholipids

The anti-inflammatory properties of highly virulent *F. tularensis* have been associated with membrane lipids enriched in this specific strain but absent in LVS [170]. An atypical phosphatidylethanolamine with a very long-chain fatty acid (C24) and a 10 carbon chain was identified as a candidate lipid, although such atypical phosphatidylethanolamine is also present in the more inflammatory subspecies *F. novicida* [171]. As phosphatidylethanolamine is the main component of the inner membrane, this anti-inflammatory lipid may quantitatively contribute to the active inhibition of innate immune responses.

#### Other virulence factors

While the T6SS is considered the most important secretion system for virulence, other secretion systems are present in *Francisella* genomes, including the TolC-dependent efflux system and type IV-pilus (Tfp), which acts as a T2-like secretion system [172].

T1SS are tripartite complexes, including the prototypical outer membrane channel of the TolC protein. TolC proteins are also outer membrane components of multidrug exporters. *Francisella* genomes contain three TolC homologues (TolC, FtlC, and SilC) that are involved in multidrug export, while the role of TolC as a T1SS component remains speculative [173,174]. TolC mutants are highly attenuated in virulence, although it is difficult to ascribe this attenuation phenotype to a deficit in multidrug efflux, a potential role in T1SS, or even a role in bacterial envelope structure [175].

T2SS relies on the Sec or Tat export system for translocation into the periplasm and allows export of proteins from the periplasm to the extracellular medium. In *F. novicida*, the closely related type IV pilus (tfp) secretion machinery acts as a T2SS-like secretion system and is involved in the secretion of several proteins including three glycosidases and a protease (PepO). PepO is absent in *F. tularensis* strains, and its role in *F. novicida* virulence is controversial [176,177]. Importantly, tfp formation can be genetically distinguished from tfp-mediated secretion. The latter appears to be more important for virulence than the tfp appendage. The tfp-exported substrates that are important for virulence remain to be identified. Although tfp-mediated secretion has only been demonstrated in *F. novicida*, the SCHU S4 strain appears to possess all the genes required for this secretion [178].

In addition, *Francisella* virulence depends on a tight control of its gene expression program, that relies on master virulence regulators including the above described MglA/SspA/PigR complex, on several transcription factors (recently reviewed [179] and two component systems (e.g. KdpD/E) [180,181] sensing various signals (oxidative stress, iron starvation, K^+^ concentration …).

Finally, numerous proteins allowing the adaptation to immune response-mediated stress are considered as virulence factors and include enzymes and proteins fighting oxidative stress (e.g. KatG [182], SodB [183], SodC [184], thioredoxinA1 [185], AhpC [184]), chaperone proteins (DsbA [186], Hfq [187]), and efflux pumps (e.g. the AcrAB system conferring resistance to antimicrobial peptides [188]).

### Immune responses

#### Innate immune responses

Innate immune responses to *Francisella* have been reviewed elsewhere [142,189] and are only briefly presented (Figure 3). As described above, owing to its particular envelope, *Francisella* is largely resistant to cationic antimicrobial peptides and to the bactericidal activity of serum components [189]. Following the first encounter with host cells, *Francisella* fully escapes TLR4 recognition and is recognized by TLR2, although, as described above, *F. novicida* limits the expression of TLR2-recognized lipoproteins. However, *F. tularensis* is much less pro-inflammatory than *F. novicida* and appears to evade TLR2 responses [33].

Following escape into the host cytosol, *Francisella* is recognized by the so-called cytosolic responses and as a first line by the cGAS/STING pathway, leading to type I IFN production [190,191]. cGAS is a cytosolic DNA sensor, suggesting that following *Francisella* escape into the host cytosol, *Francisella* genomic DNA can be detected in the cytosol, leading to IRF3 activation and type I IFN production [191]. Mice deficient in type I IFN receptor, STING, or IRF3 are more resistant to *F. novicida* infection [191,192]. Type I IFNs [193] control numerous genes during infection, and the deleterious role of type I IFN signalling for the host is likely multifactorial [194] but at least partly due to the anti-inflammatory actions of type I IFNs. Type I IFNs suppress IL-12 production by human dendritic cells following *F. tularensis* infection [195] and IL-17 production by gamma delta T cells in murine models of tularaemia .

However, type I IFN can also induce pro-inflammatory responses. Notably, type I IFN (or IFN-γ) activates IRF1 to induce Guanylate binding proteins (GBPs). GBPs target cytosolic *F. novicida* [147,196–199] to promote bacteriolysis, release bacterial DNA into the host cytosol, and activate the cytosolic DNA sensor Aim2 (Absent in Melanoma 2) in murine macrophages [190,200,201]. Aim2 is an inflammasome sensor that leads to the activation of caspase-1, release of the pro-inflammatory cytokines IL-1β and IL-18, and rapid cell death termed pyroptosis. *IRF1*, *GBP*-deficient-, and *Aim2*-deficient mice are highly susceptible to *F. novicida* infection [190,197–199]. The NLRP3 inflammasome is also important for inflammasome responses during LVS and SCHU S4 infections, although the nature of the detected stimulus remains unknown [202].

In human macrophages, GBPs are key to promoting the activation of the non-canonical caspase-4 inflammasome [147] (see above), although *F. novicida* appears to partially escape GBP targeting [203]. SCHU S4 also partially escapes GBP-mediated growth restriction and inflammasome activation [196,200,204]. IFN-γ is one of the key cytokines controlling *Francisella*-immune responses [205]. While GBPs appear to be the main effectors against *F. novicida* [200], inducible NO synthase and the produced reactive nitrogen species are key effectors against LVS [206–208] and, to a lesser extent, SCHU S4 [209,210]. In addition, IFN-γ rewires macrophage metabolism by inducing IRG-1 and producing itaconate to control intracellular replication [211].

While these pathways have mostly been described in infected macrophages, *Francisella* also infects neutrophils [48]. Neutrophils may be important cells in fighting infection [205,212]. However, they clearly contribute to host tissue damage and tularaemia pathogenesis [213]. The activation and antimicrobial activities of neutrophils are ablated by *Francisella* infection. Notably, neutrophils fail to assemble NADPH oxidase on FCP, and infected neutrophils even fail to produce reactive oxygen species in response to strong stimuli such as PMA (Phorbol 12-myristate 13-acetate), indicating that *Francisella* infection imposes a general block on neutrophil functions. The bacterial factors responsible for this impairment of neutrophil function remain unclear, although *Francisella* strains mutated in the master regulator, *fevR/pigR* or *mglA* genes are unable to block PMA-induced ROS production [213].

Despite the stealth nature of *Francisella* and its ability to dampen immune responses, the late stages of lethal infections are characterized by an unleashed immune response leading to severe sepsis. This delayed deregulated host response likely represents the cause of mortality in infected animals [214].

#### Adaptive immune responses

Adaptive immune responses to *Francisella* spp. have recently been reviewed [215]. Surprisingly, for intracellular pathogens, antibodies have been found to be protective against tularaemia [216]. Notably, anti-LPS IgMs generated in a timely manner by B1a B cells in the peritoneal cavity are protective in passive immunization experiments [217]. Both CD4 and CD8 T cells are important factors in the *Francisella* immune response, notably through the production of IFN-γ and TNF [218]. While IFN-γ is highly bactericidal in macrophages infected with LVS and *F. novicida* [200,219], *F. tularensis* appears to escape at least partially IFN-γ-mediated restriction, and macrophages require both TNF and IFN-γ to control infection [210,220].

## Conclusions

Tularemia is currently a rare disease. Yet, it remains a concern in many countries because of its poorly understood ecological dynamics and high infectivity of *F. tularensis* through aerosol transmission. Although *Francisella* is a zoonotic pathogen and may not have evolved with the human host, it evades detection by the innate immune system. This stealth ability appears to be related to its lipopolysaccharide, envelope, and capsule, which are poorly immune-activating. Recently, the study of *Francisella* has shed new light on its main virulence factors, the T6SS, and its effectors, although the central mechanism of phagosomal rupture is still not well understood. Additionally, *Francisella* appears to hijack host cell metabolism to promote massive replication within the host cytosol. Undoubtedly, future research on *Francisella* will continue to reveal new and original bacterial mechanisms for replicating within host cells and causing diseases.

## Data Availability

The data, materials and original svg figures that support the results or analyses presented in this paper are freely available upon request.
